# Excitatory-inhibitory tone shapes decision strategies in a hierarchical neural network model of multi-attribute choice

**DOI:** 10.1371/journal.pcbi.1008791

**Published:** 2021-03-11

**Authors:** Warren Woodrich Pettine, Kenway Louie, John D. Murray, Xiao-Jing Wang

**Affiliations:** 1 Center for Neural Science, New York University, New York, United States of America; 2 Department of Psychiatry, Yale University School of Medicine, New Haven, United States of America; Ecole Normale Superieure, FRANCE

## Abstract

We are constantly faced with decisions between alternatives defined by multiple attributes, necessitating an evaluation and integration of different information sources. Time-varying signals in multiple brain areas are implicated in decision-making; but we lack a rigorous biophysical description of how basic circuit properties, such as excitatory-inhibitory (E/I) tone and cascading nonlinearities, shape attribute processing and choice behavior. Furthermore, how such properties govern choice performance under varying levels of environmental uncertainty is unknown. We investigated two-attribute, two-alternative decision-making in a dynamical, cascading nonlinear neural network with three layers: an input layer encoding choice alternative attribute values; an intermediate layer of modules processing separate attributes; and a final layer producing the decision. Depending on intermediate layer E/I tone, the network displays distinct regimes characterized by linear (I), convex (II) or concave (III) choice indifference curves. In regimes I and II, each option’s attribute information is additively integrated. In regime III, time-varying nonlinear operations amplify the separation between offer distributions by selectively attending to the attribute with the larger differences in input values. At low environmental uncertainty, a linear combination most consistently selects higher valued alternatives. However, at high environmental uncertainty, regime III is more likely than a linear operation to select alternatives with higher value. Furthermore, there are conditions where readout from the intermediate layer could be experimentally indistinguishable from the final layer. Finally, these principles are used to examine multi-attribute decisions in systems with reduced inhibitory tone, leading to predictions of different choice patterns and overall performance between those with restrictions on inhibitory tone and neurotypicals.

## Author summary

In a multi-attribute decision task under environmental uncertainty, a multi-layer neural network’s E/I tone can shape sophisticated strategies for selection of high-valued offers. These strategies result from a inhibition-driven tuning of time-varying nonlinear computations in intermediate layer areas. This suggests the degree of signal linearity observed brain areas during decision tasks is a direct result of task structure. Furthermore, the performance of subjects with restrictions on inhibitory tone and neurotypicals will differ on a multi-attribute decision task as uncertainty increases.

## Introduction

When choosing between cereals on a grocery store shelf, one might consider multiple attributes, such as each alternative’s flavor or healthiness. Systems neuroscientists have, for many years, been studying the specific circuits engaged in this kind of multi-attribute decision-making. Based on a robust set of electrophysiology and imaging findings [1–8], many hold that all attribute signals are available in brain areas proximal to the final decision [9, 10]. However, an alternative perspective has recently arisen taking into account the multiple brain areas implicated in decisions [11–18]. Under this alternate view, a signal is transformed as it moves from hierarchically lower areas to those that are proximal to the final decision [19–21]. For example, individual attribute values can be coded in the orbitofrontal cortex (OFC), as modulated by the timing of presentation or the offers present, prior to integration as a choice signal in the dorsolateral prefrontal cortex (dlPFC) [22]. It is hypothesized this process can allow for parallel computation of individual values or actions, and may produce a clearer separation between representations of choice alternatives where the decision is reached, yet a rigorous mechanistic description is lacking. A mechanistic description is all the more important as transformations along a hierarchy can be highly nonlinear, providing an additional layer of flexibility when engaging in sophisticated choices.

Such flexibility is essential in real-world environments, where the true attribute values are unlikely to be precisely known. For example, when deciding between grocery store cereals, their exact flavor or healthiness might be unclear. Uncertainty as to values and outcomes has been a useful concept in studying decision behavior [23]. Furthermore, environmental uncertainty has been utilized in the study of many neuropsychiatric conditions, such as autism spectrum disorder (ASD) and schizophrenia (SCZ) [24–29]. A mechanistic understanding of how hierarchical neural systems handle environmental uncertainty when making decisions can not only provide knowledge as to how the brain functions, but also may produce mechanistic insights into neuropsychatric disorders.

Prior dynamical multi-attribute models have accounted for a number of counter-intuitive behavioral phenomena [30, 31]. These insightful models, however, utilized mathematical operations specific for the psychological phenomena they captured. The question remains, how are attribute signals processed in the brain, and in what ways are these computations constrained by neural operations?

If one is to investigate the non-linear transformations that can occur in populations of neurons during such tasks, it is imperative to consider both the time dynamics of neural activity, as well as excitatory and inhibitory (E/I) tone. Time dynamics have been shown to be an essential feature of neural computations used in value coding [18, 32–37], while the various effects of E/I tone have been studied extensively. In sensory areas, E/I tone can shape the stimulus tuning curve and responses timing [38, 39]. In perceptual decision-making, E/I tone can dictate the trade-off between speed and accuracy [45]. In working memory tasks, E/I tone defines a network’s ability to maintain a memory, and that memory’s susceptibility to a distractor [40, 41]. When multiple areas interact, the E/I tone can define their function in either memory maintenance or decision-making [42]. On a more theoretical level, E/I tone can shape a system’s response as an input signal increases [43] and its transition to chaotic dynamics [44]. E/I tone has also been a useful framework for describing brain-wide changes in disorders such as schizophrenia and ASD [25]. Yet, it remains to be described how E/I tone defines the functional interaction and transformation of multiple time-varying signals passing through brain areas. This is all the more critical, given the array of areas currently implicated in decision-making, and the rapidly improving large-scale recording technologies.

We use a three-layer, feed-forward network of dynamical cascading nonlinearities parameterized by E/I tone to investigate the basic decision properties of a hierarchical system. The Hierarchical Network contains an input layer of value-coding neurons, an intermediate layer for signal transformation, and a final winner-take-all layer from which decisions are read out. The intermediate and final layers are composed of neural units with recurrent excitation and cross-inhibition that are specific for attributes or offers. Utilizing analytical tools from economics, we find that the E/I tone of the intermediate layer creates distinct regimes defined by their choice indifference curve: a linear weighting of attribute values (regime I); a convex preference for balanced attributes (regime II); or a concave preference with increased weighting of the larger attribute value (regime III). These decision regimes are primarily driven by the level of inhibition. We then compare the performance of the Hierarchical Network to a conventional Linear Network on a multi-attribute decision task under environmental uncertainty. When uncertainty is low, the Linear Network and regime I of the Hierarchical Network—where the choice area has access to all signal information—more consistently choose the larger-valued alternative. However, when uncertainty is high, the Hierarchical Network’s regime III—with strong inhibition-driven nonlinear transforms along the hierarchy—improves in relative performance. We show this shift in relative performance is primarily due to regime III weighting the attributes by salience. This salience effect involved an increased separation of offer distributions by a winner-take-all function, and a dynamically emergent selective attending to attributes by their difference across alternatives. Closely examining readout from the intermediate layer, we find that one can consistently read out decision signals with great accuracy, even in areas without access to all attribute information. The hierarchical framework is then used to create a model of neurotypicals with the full range of E/I tone, and a model of populations with limited inhibitory tone. Based on the models, we make the novel prediction that neurotypical subjects and those with limited inhibitory tone will adopt a linear weighting of attributes in environments with low uncertainty (regimes I/II). As environmental uncertainty increases, neurotypical subjects will fully move into regime III, while restricted inhibitory tone individuals will not. This will result in larger performances differences at high environmental uncertainty levels.

## Materials and methods

### Cortical areas

Single areas in the networks were composed of mean-field attractors that have been used to model working memory, perceptual decision-making, as well as to model individual areas in multi-area cortcal simulations [42, 45–48]. Each area consisted of two populations, such that *c* = *A*, *B*, where each population was associated with a choice alternative. The dynamics of a given population is described by a single synaptic gating variable representing the fraction of activated N-methyl-D-aspartate receptor (NMDA) conductance and governed by the equation [45],

$$\begin{array}{c} \frac{dS}{dt}=-\frac{S}{\tau_{NMDA}}+\gamma \left(1-S\right)r, \end{array}$$ (1)

where the NMDA time constant *τ*_*NMDA*_ = 60 ms, and the rate of saturation is controlled by *γ* = 0.641. The firing rate *r* was a function of the input current *I*, as defined by the curve relation first described in [49]:

$$\begin{array}{c} r=F\left(I\right)=\frac{aI-b}{1-exp[-d(aI-b)]}, \end{array}$$ (2)

where *a* = 270 Hz/nA, *b* = 108 Hz, and *d* = 0.154 seconds. The total synaptic current consisted of recurrent (*I*_*rec*_), noisy (*I*_*noise*_), background (*I*_*o*_ = 0.3297 nA) and external components such that,

$$\begin{array}{c} I=I_{rec}+I_{noise}+I_{o}+gI_{input}, \end{array}$$ (3)

where the coupling constant *g* = 0.0011 nA/Hz converts the firing rate *I*_*input*_ to current. As described in section 0.2, specific values of *I*_*input*_ are notated as *I*_*A*,1_, *I*_*A*,2_, *I*_*B*,1_, or *I*_*B*,2_. For a given population *i* in area *n* of the the network, the recurrent current was given by the equation,

$$\begin{array}{c} I_{rec,i}^{n}=\sum_{m,j}S_{j}^{m}J_{ij}^{\left(m\to n\right)}, \end{array}$$ (4)

where $J_{ij}^{\left(m\to n\right)}$ is the strength of the connection from population *j* in area *m* to population *i* in area *n*. The use of this equation in the Linear Network and Hierarchical Network is described in the following sections.

The noise currents for each population were defined by an Ornstein-Uhlenbeck process:

$$\begin{array}{c} \tau_{AMPA}\frac{dI_{noise}\left(t\right)}{dt}=-I_{noise}\left(t\right)+\eta_{I}\left(t\right)\sqrt{\tau_{AMPA}\sigma_{noise}^{2}}, \end{array}$$ (5)

where the time constant *τ*_*AMPA*_ = 2 ms, and *η*_*I*_ is a Gaussian white noise term with mean zero, and variance $\sigma_{noise}^{2}=0.003\;\text{nA}$.

### Inputs

External inputs were provided as firing rates, spanning the range of 0 to 40 Hz. Notationally, they are given by *I*, such that the input of attribute 1 for choice alternative *A* was written *I*_*A*,1_, etc. An unspecified choice alternative is indicated with the population subscript “*c*” and unspecified attribute by the subscript “*x*.”

When varying environmental uncertainty, a value *η*_*I*_ was randomly drawn from $N(0,\sigma_{\eta_{I}}^{2})$ independently for each attribute on each trial, such that *I*_*c*,*x*_ = *I*_*c*,*x*_ + *η*_*I*_. The variance $\sigma_{\eta_{I}}^{2}$ was used to control the amount of environmental uncertainty in a given block of trials.

In several places, we refer to a normalized value of the input, ${\hat{I}}_{c,x}$. In those cases, we specify the quantity used in normalization.

### Linear network

The Linear Network consisted of two layers. The first layer provided inputs from the individual attributes, in the form of firing rates *I*_*input*_. The attribute inputs “*a*” to a given population were linearly summed, such that

$$\begin{array}{c} I_{c}=w\sum^{a}I_{c,a}, \end{array}$$ (6)

where *w* = 0.5. These values were then fed into the final area described in section 0.1. As there was only one area, Eq 4 simplified to,

$$\begin{array}{c} I_{i}=\sum_{j}S_{j}J_{ij}, \end{array}$$ (7)

where for *i* = *j*, the connection strength was 0.3725 nA, and where *i* ≠ *j* the connection strength was −0.1137 nA [45]. The network choice was determined when one of the final area populations passed the firing rate threshold of 35 Hz.

### Hierarchical networks

The input layer to the Hierarchical Networks was also specified by *I*_*c*,*x*_. In the Hierarchical Network, inputs were segregated by attribute then transmitted to an intermediate transform layer, consisting of two-population areas specific for each attribute. These areas were structured along the lines described in section 0.1.

In the intermediate layer areas of the Hierarchical Network, the connection strengths in Eq 4 were defined as follows. *J*_+_ controlled the strength of the excitatory connection of a population to itself (*A* → *A*, etc.), and *J*_−_ the strength of the inhibitory connection from one population to another (*A* → *B*, etc.). The strength of *J* between areas in the intermediate layer areas was set to 0. Connectivity from the intermediate transform layer (TL) areas to the final layer (FL) was restricted to excitation between populations with the same selectivity, such that $J_{AA}^{\left(TL\to FL\right)}=0.25$, $J_{AB}^{\left(TL\to FL\right)}=0.0$, etc.

The excitatory and inhibitory weights in this intermediate layer (*J*_+_ and *J*_−_) were parametrically varied to achieve the specific Hierarchical Network E/I tones described in the Results section. For simplicity, weights in all areas in the processing layer were symmetrically varied, such that when the recurrent excitation or cross-inhibition were changed, all areas in the processing layer assumed the specified values. The outputs from this processing layer were continuously fed into a final area of the same type as the final area of the Linear Network in section 0.1. To clarify notation, when an input *r* is passed through an intermediate transform layer area, its associated transformed output is referred to as *T*. The value *T* is that of the synaptic gating variable from Eq 1.

### Simplified non-dynamical models

We also used non-dynamical linear and max operations to investigate the separation of offer distributions. These models used the input values and uncertainty level to create distributions of decision values.

#### Linear operation

The linear operation involved a simple linear sum of the attribute input distributions such that,

$$\begin{array}{c} DV_{c}=N\left(I_{c,1}+I_{c,2},2\sigma_{\eta_{I}}^{2}\right) \end{array}$$ (8)

#### Max operation

The max operation was conducted in the attribute space, such that only the larger attribute was included in the decision value. For each attribute, the lower attribute value among the two alternatives is ignored (set to 0). In case of equality, the one set to 0 is chosen randomly. Then, alternatives are compared based on their overall value, which is the sum of their (max-transformed) attribute values. Thus, depending on the outcome of that max operation, the final decision layer would choose between values drawn from the following sets of distributions:

if *I*_*A*,1_ > *I*_*B*,1_ and *I*_*A*,2_ > *I*_*B*,2_,

$$\begin{array}{c} \begin{array}{c} \begin{array}{cc} DV_{A} & =N(I_{A,1}+I_{A,2},2\sigma_{\eta_{I}}^{2})\\ DV_{B} & =0; \end{array} \end{array} \end{array}$$ (9a)

if *I*_*A*,1_ > *I*_*B*,1_ and *I*_*A*,2_ < *I*_*B*,2_,

$$\begin{array}{c} \begin{array}{c} \begin{array}{cc} DV_{A} & =N(I_{A,1}+,\sigma_{\eta_{I}}^{2})\\ DV_{B} & =N(I_{B,2},\sigma_{\eta_{I}}^{2}); \end{array} \end{array} \end{array}$$ (9b)

if *I*_*A*,1_ < *I*_*B*,1_ and *I*_*A*,2_ > *I*_*B*,2_,

$$\begin{array}{c} \begin{array}{c} \begin{array}{cc} DV_{A} & =N(I_{A,2}+,\sigma_{\eta_{I}}^{2})\\ DV_{B} & =N(I_{B,1},\sigma_{\eta_{I}}^{2}); \end{array} \end{array} \end{array}$$ (9c)

or if *I*_*A*,1_ < *I*_*B*,1_ and *I*_*A*,2_ < *I*_*B*,2_,

$$\begin{array}{c} \begin{array}{c} \begin{array}{cc} DV_{A} & =0\\ DV_{B} & =N(I_{B,1}+I_{B,2},2\sigma_{\eta_{I}}^{2}). \end{array} \end{array} \end{array}$$ (9d)

### Model behavior

#### Psychometric curves

For the psychometric curves, input values associated with alternative *A* were varied from 15 to 25 Hz, (step size of 0.5 Hz) while those associated with alternative *B* were held constant at 20 Hz. 1,000 trials were run for the Linear Network, and for the Hierarchical Network. We then calculated the percent of those trials where the networks chose the option associated with the varied attribute. To these points, we fit a sigmoid of the form:

$$\begin{array}{c} P\left(c\right)=\frac{1}{1+exp(-kv-\mu ))}, \end{array}$$ (10)

where *P*(*c*) represents the proportion of the time the varied choice alternative was chosen, *k* dictates the slope of the sigmoid, *v* is calculated from $\left({\hat{I}}_{A,1}+{\hat{I}}_{A,2}\right)-\left({\hat{I}}_{B,1}+{\hat{I}}_{B,2}\right)$, and *μ* provides the centering of the sigmoid. The normalized values ${\hat{I}}_{A,2}$, etc. were determined by setting to 0.5 the attribute values associated with alternative *B*, and then accordingly scaling values associated with alternative *A*.

#### 0.3.1 Indifference curves

The indifference curves were calculated by independently varying the values for choice alternative *A* (*I*_*A*,1_ and *I*_*A*,2_), while holding *I*_*B*,1_ and *I*_*B*,2_ constant as a reference. The reference attribute inputs were both 20 Hz, and the varied attribute inputs ranged from 0 Hz to 40 Hz. All possible combinations of the varied attributes were then simulated for 1,000 trials, where the networks chose between the varied alternative and the reference. This was done for the Linear Network, as well as for several processing-layer E/I tones of the Hierarchical Network. The intermediate layer transform area E/I tones were all possible combinations of the recurrent excitatory weights 0.30 to 0.40 nA, spaced 0.01 nA with the cross-inhibitory weights 0.0 to −0.1 nA, spaced 0.01 nA. Indifference values were then taken to be those where the network chose evenly between the alternatives.

To fit an indifference curve, we first normalized the indifference values (and associated inputs) such that the minimum in either direction was 0, and the maximum was 1. That gave us $\hat{I}$. We then used a constant elasticity of substitution utility equation [50] of the form,

$$u({\hat{I}}_{A,2},{\hat{I}}_{A,2})={\left({\hat{I}}_{A,1}^{a}+{\hat{I}}_{A,2}^{a}\right)}^{1/a}.$$ (11)

By setting the utility to 1 and solving for ${\hat{I}}_{A,2}$ as a function of ${\hat{I}}_{A,1}$, we arrived at the form,

$${\hat{I}}_{A,2}={\left(1-{\hat{I}}_{A,1}^{a}\right)}^{1/a},$$ (12)

where *a* was used to define the shape of the indifference curve. This equation was fit to the data using the curve_fit function from scipy [51]. If *a* < 1, the curve was classified as convex. If 1 < = *a* < = 1.2, it was classified linear, and if *a* > 1.2 it was classified as concave. These classifications were then used to partition the weight space into areas that produced convex, linear or concave indifference curves.

#### 0.3.2 Multi-attribute decision task offers

To simulate the multi-attribute decision task, we created sets of offers and presented them as firing rate inputs to the network. For the first set of simulations, we took an array of values 10 to 20 Hz, with a step size of 2 Hz, and then combinatorially assigned those values to *I*_*A*,1_, *I*_*A*,2_, *I*_*B*,1_, and *I*_*B*,2_. This led to 630 possible offers.

We then narrowed the selection of offers to specific quadrants of the indifference space. To do this, we set a reference of 15 Hz and then created two vectors: one from 10 to 15 Hz, and a second from 15 to 20 Hz, both spaced by 0.5 Hz. We used these to sample from the space, eliminating any cases where the values of the alternatives were equal. This led to 91 offers.

#### Comparison of network task performance

To quantify performance on the task, we looked at the proportion of trials on each offer set where a network chose the larger of the alternatives, denoted as *P(Larger Chosen)*. Trials where the network failed to reach the decision threshold were included in denominator when calculating the proportion larger chosen. We excluded offer sets where the two alternatives were equal.

We ran this task on the Linear Network, as well as the Hierarchical Network, with all combinations of the recurrent excitatory weights ranging from 0.30 nA to 0.36 nA, spaced 0.01 nA, with the cross inhibitory weights ranging from 0.0 nA to −0.1 nA, spaced 0.01 nA. Each choice was run for 1,000 trials, from which the proportion of choices for alternative *A*, alternative *B*, and failed trails was recorded.

When comparing the performance of the network frameworks, we examined the proportion of offers where the Hierarchical Network was more likely than the Linear Network to choose the larger offer. We could have instead looked at the proportion of total trials where each chose the larger offer, as was done in other analyses. The use of offer proportions in comparing the frameworks (rather than overall trials) was a more conservative measure, as the Hierarchical Network could have simply been performing extremely well on a minority of offers at the expense of the majority of offers.

#### Best performing E/I tone and indifference curve curvature

To determine the best performing weight structures and their associated indifference curvatures, we looked at the Hierarchical Network E/I tone that maximized the *P(Larger Chosen)* metric, as well as its associated indifference curvature value. To these values, we then fit sigmoids of the form,

$$\begin{array}{c} P\left(c\right)=\frac{a}{1+exp(-kv-\mu ))}+c, \end{array}$$ (13)

where *a* determined the saturation point of the sigmoid and *c* it’s intercept.

#### Intermediate layer readout

When reading out timing or decision results from the intermediate layer transform areas, we utilized a within-attribute threshold crossing metric, first described in [42]. For each intermediate layer transform area, we examined the firing rates of each population, and then recorded the time where their firing rate difference crossed a threshold of 12 Hz, as well as the population with the higher firing rate. If the threshold failed to be crossed prior to a decision by the final area, it was marked as a failed trial for that intermediate layer transform area. When calculating the threshold crossing difference, we took the absolute value of the time difference for threshold crossing between areas. If one of the populations failed to cross the threshold, the time difference was given between the successful population and the final area decision time.

To read out a decision from an intermediate layer area, we looked at population with the highest firing rate at the earliest threshold crossing. If the threshold failed to be crossed by any intermediate layer transform area prior to a decision by the final area, it was marked as a failed trial.

### Fixed point analysis

To compute the fixed points and phase portraits of a processing layer area, we used the python package pydstool [52], parameterized by the equations described in 0.1, with the internal noise set to 0. We first defined an attribute input as a coordinate in euclidean space space whose positions are defined by the level of each input. We then defined a phase plane by applying these inputs to each E/I tone configuration. A starting position in each phase plane was selected near the origin at (0.06, 0.06), and a noiseless trajectory was computed. The end point of that trajectory was taken as the output of the transform layer.

#### Fixed points fit to full model behavior

To test if fixed point trajectories well-approximated the behavior of the full network, we used psychometric functions with the independent variable derived from the fixed points, and the dependent variable from the proportion of *A* chosen by the full network. We ran both the fixed point trajectories and the full network for all values described in section 0.3.1. The trajectory end point values were treated as approximations of the *T* values and summed to created the choice value, such that *F*_*A*_ = *T*_*A*,1_ + *T*_*A*,2_ and *F*_*B*_ = *T*_*B*,1_ + *T*_*B*,2_. If these approximations represent the transforms performed by the full network, the network should choose *A* whenever *F*_*A*_ > *F*_*B*_, regardless of indifference curve shape. In such a case, the *P*(*A*) from the network should track the *F*_*A*_ − *F*_*B*_ derived from fixed points trajectory endpoints. We therefore fit Eq 10, with *v* = *F*_*A*_ − *F*_*B*_ from the trajectories and *P*(*A*) from the full network, and used *R*^2^ as a measure of how well the fixed points predicted choice behavior of the full network.

#### Calculation of convexity

The trajectory endpoints were computed for all inputs and E/I tones described in section 0.3.1. These endpoints were treated as the values *T*. They were then linearly summed to determine the value of a choice alternative, such that,

$$\begin{array}{c} F_{c}=\sum_{a}T_{c,x}. \end{array}$$ (14)

The indifference point was taken to be when the *F*_*A*_ ≈ *F*_*B*_. Convexity was then computed and the weight space was partitioned using the methods described in section 0.3.1.

#### Decision-threshold

The intermediate layer transform area decision threshold was determined by setting Eq 1 to 0, then solving for the one synaptic variable as a function of the other, providing the equation,

$$\begin{array}{c} \begin{array}{c} S_{1}=(\gamma \tau (r_{thresh}+\frac{S_{2}}{r_{thresh}+\gamma \tau \left(1-S_{2}\right)}))/\\ (1+\gamma \tau (r_{thresh}+\frac{S_{2}}{r_{thresh}+\gamma \tau \left(1-S_{2}\right)})). \end{array} \end{array}$$ (15)

### Neurotypical and restricted inhibitory tone

We introduced a module that assessed the level of uncertainty in the environment, then used a continuous function to set the E/I tone in the intermediate layer areas to a tone that optimized performance. To do this, we ran the task on all E/I tones configurations as described in Section 0.3.2. Given the extensive run-time associated with simulations, we selected the E/I tone closest to that given by the continuous function. The neurotypical model was able to utilize the full range of excitation and inhibition. The limited inhibitory tone model was able to utilize the full range of excitation, but the inhibitory tone was limited to a maximum of −0.01 nA. We then presented the models with blocks of trials at different uncertainty levels, and calculated the *P(Larger Chosen)* (section 0.3.2) during each block.

## Results

### Hierarchical networks adopt distinct decision regimes dictated by intermediate layer E/I tone

To investigate the behavior of multi-layer hierarchical neural networks, we used a multi-attribute decision task. On any given trial, the networks were presented with two choice alternatives (*A* or *B*). These choice alternatives were each composed of two attributes (1 or 2). For simplicity, attributes were assumed to have equal contributions to subjective value and action costs were assumed to zero. When indexing, the first value refers to the choice alternative, and the second to the attribute. Thus, choice alternative *A* was presented to the network using input *I*_*A*,1_ and input *I*_*A*,2_, while choice alternative *B* was presented to the network using input *I*_*B*,1_ and input *I*_*B*,2_. Inputs were translated from offer values to firing rates. As competition within the final area was between alternatives, the signal at that stage is denoted by *F*_*A*_ or *F*_*B*_, where the subscript indicates the choice alternative. This integration of value attributes into a decision signal is similar to that recently observed in the dlPFC [22]. On a given trial, the choice between *A* or *B* was determined by the first population in the final area (*F*_*A*_ or *F*_*B*_) to cross a firing rate threshold (35 Hz).

**Fig 1:**
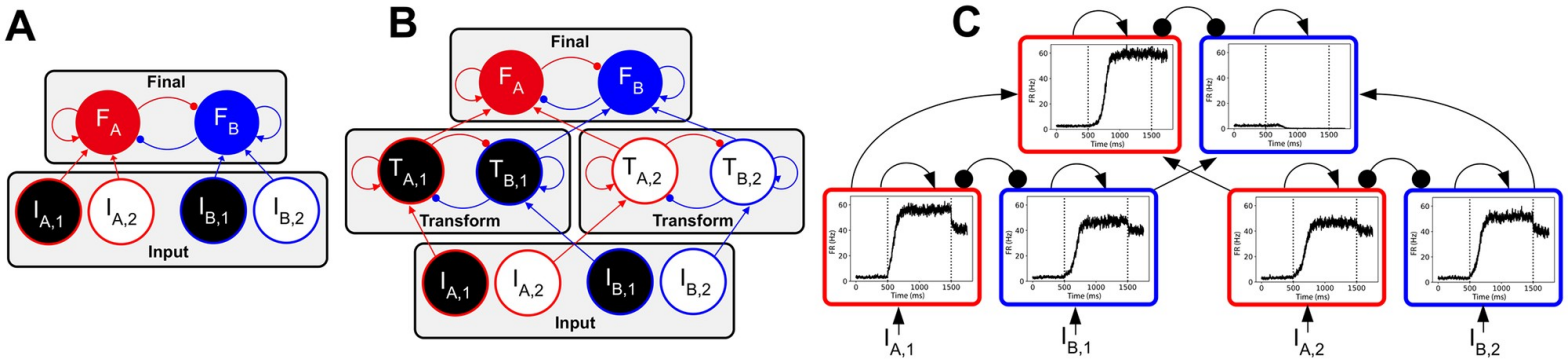
Network schematics and sample trial. *I*: input; *F* final layer synaptic gating variable; *T*: intermediate transform layer synaptic gating variable; red: alternative *A*; blue: alternative *B*; black: attribute 1; white: attribute 2; arrows: excitation; circles: inhibition. Both networks include an input layer and a final layer. (A) The Linear Network consists of two layers, with attribute signals from the input layer directly transmitted to the final layer. (B) The Hierarchical Network includes an additional intermediate layer that performs a functional transformation on the attribute signals prior to their passing to the final area. (C) A sample trial of the Hierarchical Network, with intermediate layer weights *J*_+_ = 0.34 nA and *J*_−_ = −0.02. For each area, the X-axis indicates time, while the Y-axis indicates the population firing rate. The first vertical line indicates the onset of the offer value signal, and the second vertical line indicates termination of the offer value signal.

We created two basic network frameworks: the Linear Network and the Hierarchical Network. Areas in these networks were composed of mean-field approximations of population firing rates, parameterized by recurrent excitation and cross-inhibition (see Materials and methods). As the name suggests, the Linear Network computed an exact linear weighted sum of the attributes (weight = 0.5). Thus, with the Linear Network, the final area received a signal that was linearly translated from the offer values (Fig 1A). The Linear Network has a similar architecture to that presented in [53], and represents the case where all attribute signals are fully available to a final area.

Several prior computational models have proposed parallel processing of attributes [30, 31, 54–58]. Furthermore, recent studies involving humans directly examined attribute-specific processing prior to integration in downstream areas [59–61]. Therefore, in the Hierarchical Network, inputs were first transformed by intermediate, attribute-specific areas (Fig 1B). The intermediate layer transforming the input signal was a nonlinear dynamical system, composed of mean-field populations associated with each choice alternative (see Materials and methods). To denote the transformation performed by an intermediate layer transform area on the input signal, its output signal will be referred to as *T*, such that *I*_*A*,1_ is related to *T*_*A*,1_, etc. These continuous output signals were integrated by the final winner-take-all area that determined the decision on a given trial. A single trial of the Hierarchical Network is shown in Fig 1C. This framework, similar in structure to that presented in [56], represents the simplest form of a network with parallel processing streams, where transformations are performed on attribute signals passing through specialized areas prior to the final decision.

**Fig 2:**
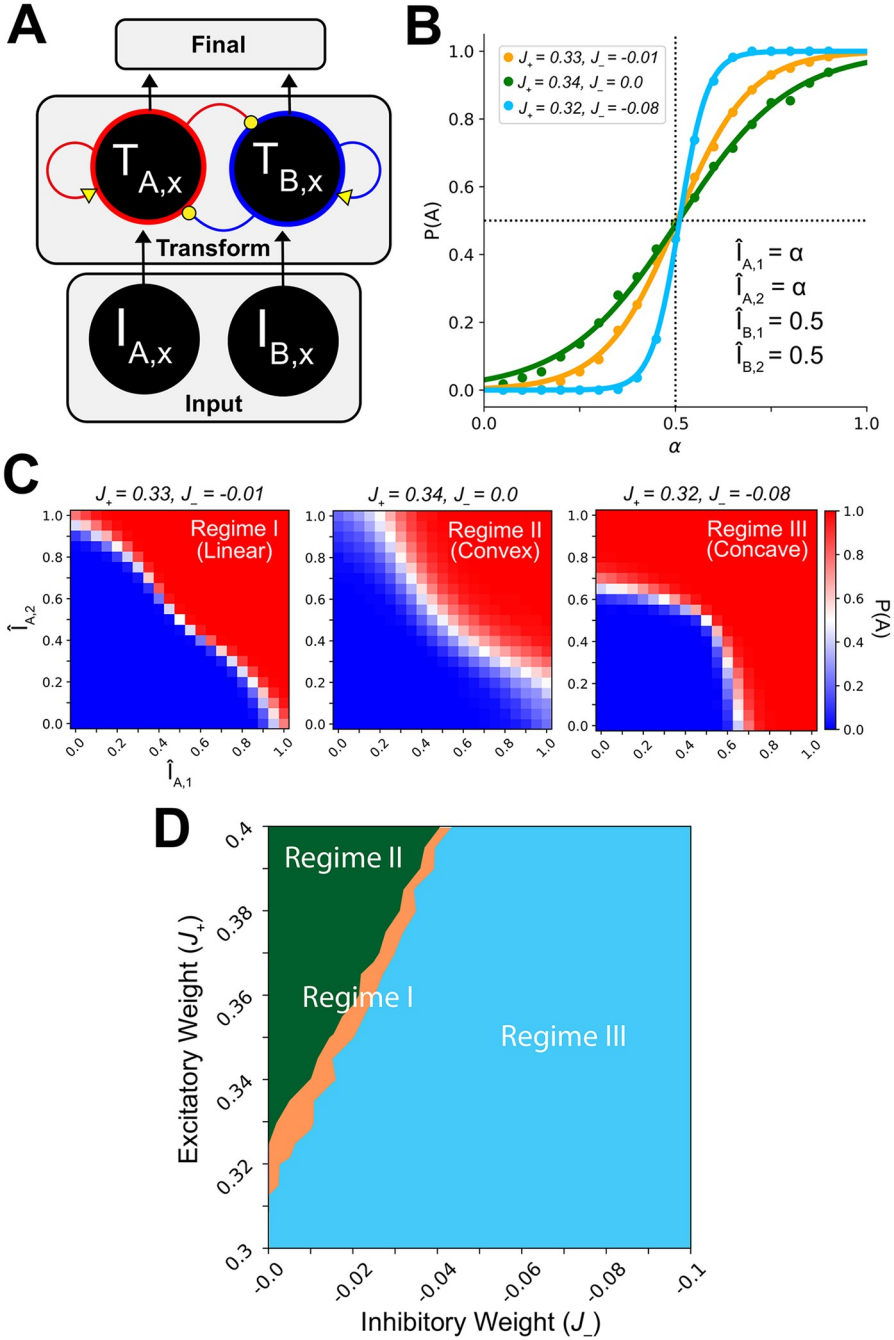
Hierarchical networks produce distinct decision regimes. *I*: input; $\hat{I}$: input normalized by the reference choice alternative values’ linear sum; *T*: transform layer synaptic gating variable; *P*(*A*): proportion of 1,000 trials where alternative A was chosen; *J*_+_: excitatory weight; *J*_−_: inhibitory weight; yellow: varied weights; forest green: regime II; tan: regime I; sky blue: regime III. The first subscript indicates choice *A* or *B*, and the second subscript *x* indicates a generic attribute. Weights were systematically varied identically in all intermediate layer areas. (A) A diagram of a generic intermediate layer area and its inputs. (B) The Hierarchical Network produced expected psychometric functions. (C) Modification of intermediate layer E/I tone produced distinct indifference curve regimes. Examples of regime I (linear), regime II (convex) and regime III (concave) decision regimes. (D) The space of E/I tones can be partitioned by decision regime according to their indifference curves.

To verify that the Hierarchical Network was able to perform the task, we computed simple psychometric functions of choice behavior. This was done by linearly increasing the input values associated with choice alternative *A* (*I*_*A*,1_ and *I*_*A*,2_), while keeping inputs associated with *B* (*I*_*B*,1_, and *I*_*B*,2_) fixed as a reference. We then varied the intermediate transform layer excitatory and inhibitory weights (colored gold in Fig 2A) and measured the proportion of trials that networks chose *A* (referred to as *P*(*A*)). A sigmoid function was fit to the resultant *P*(*A*) (see Materials and methods). Fig 2B shows the results for three intermediate transform layer E/I tone configurations. The networks were able to generate expected psychometric functions, increasingly choosing *A* as its offer value increased.

Having verified the Hierarchical Network is able to perform the task, we next examined how it utilizes attribute information. While the Linear Network performs a simple linear sum of the attributes, the Hierarchical Network first passes the input signals through attribute-specific intermediate areas parameterized by E/I tone (Figs 1B and 2A).

To investigate how the transform of attribute inputs changed with E/I tone, we used indifference curves. The Linear Network by definition computes a weighted sum of the attributes, so its indifference curve is trivially linear, following the line where *I*_*A*,1_ + *I*_*A*,2_ = *I*_*B*,1_ + *I*_*B*,2_. For the Hierarchical Network, however, the indifference curve provides a tool for analyzing how it weighs and combines attribute information (Fig 2). A linear indifference curve indicates that the network is performing a linear combination (similar to that of the Linear Network). A convex indifference curve indicates that the network places greater weight on attribute values composing a choice alternative that is balanced between the attributes, and less weight on attribute values composing an unbalanced choice alternative. A concave indifference curve indicates the opposite: specifically, the network underweighs the smaller attribute value, and instead places preferentially greater weight on the salience of larger attributes.

When generating an indifference curve for each E/I tone, we again held *I*_*B*,1_ and *I*_*B*,2_ constant as a reference. We then independently varied the magnitudes of *I*_*A*,1_ and *I*_*A*,2_ (see Materials and methods). For each offer, we calculated the proportion of 1,000 trials where the network chose alternative A, producing *P*(*A*). For some values of *I*_*A*,1_ and *I*_*A*,2_, (for example, when both are much less than their counterparts) *P*(*A*) will be low. For other values of *I*_*A*,1_ and *I*_*A*,2_, (for example, when both are much greater than their counterparts) *P*(*A*) will be high. However, there will be a set of values of *I*_*A*,1_ and *I*_*A*,2_ where the subject is indifferent between the choices and *P*(*A*) ≈ 0.5. The values where *P*(*A*) ≈ 0.5 were used to fit the indifference curve.

We parametrically varied the levels of excitatory (0.30 nA to 0.40 nA, spaced 0.05 nA) and inhibitory weights (0.0 nA to −0.10 nA, spaced 0.05 nA) within intermediate layer transform areas (Fig 2A). For each E/I tone configuration, we presented the offers and then fit a single-parameter exponential function to the points where *P*(*A*) ≈ 0.5 (see Materials and methods). That single parameter determined the curvature, such that a value of 1 indicates a linear indifference curve, a value less than one indicates convex, and a value greater than 1 indicates concave. We found that at different levels of excitation and inhibition, the network adopted distinct decision regimes, corresponding to linear (regime I), convex (regime II) and concave (regime III) indifference curves (Fig 2C). The appearance of these linear-input decision regimes formed distinct regions, such that we were able to use the regimes to partition the space of E/I tones (Fig 2D). This revealed that by configuring the levels of excitation and inhibition in the intermediate layer, the Hierarchical Network can adopt distinct decision-making regimes.

### Regime III arises from a winner-take-all operation

Having established that the E/I tone can define decision-making regimes, we next examined the functional transformation taking place within an intermediate layer attribute-specific transform area.

The phase planes of dynamical systems are a highly useful tool for analyzing the functional properties of decision networks [45, 46]. For an intermediate layer transform area in the Hierarchical Network, each combination of E/I tone and input produces a unique phase plane. We calculated phase planes for all combinations of an intermediate layer transform area’s excitatory tone (0.30 nA to 0.40 nA, spaced 0.05 nA) and inhibitory tone (0.0 nA to −0.10 nA, spaced 0.05 nA), and all input combinations of 0 to 40 Hz, spaced 0.5 Hz. This produced 793,881 total phase planes. For each phase plane, we computed a noiseless trajectory starting near the origin. The endpoint of that trajectory was taken as the output of the specific combination of network structure and inputs. We used these fixed point trajectory endpoints to approximate *T*_*A*,1_, *T*_*A*,2_, *T*_*B*,1_, and *T*_*B*,2_. The decision was determined by linearly summing these *T* values to obtain *F*_*A*_ and *F*_*B*_, then choosing the maximum of these two choice values.

Though the intermediate layer outputs to the final area in the full dynamical model often did not reach the fixed points (due to the decision threshold), the fixed points did a reasonable job of approximating behavior. To measure this, for each E/I tone we fit a sigmoid to the choice behavior of the full model as a function of the difference between choice alternative fixed points (see Materials and methods). As over 90% of E/I tone configurations had an *R*^2^ > 0.70, we were confident in the use of fixed points as an approximation for analysis of intermediate layer function.

**Fig 3:**
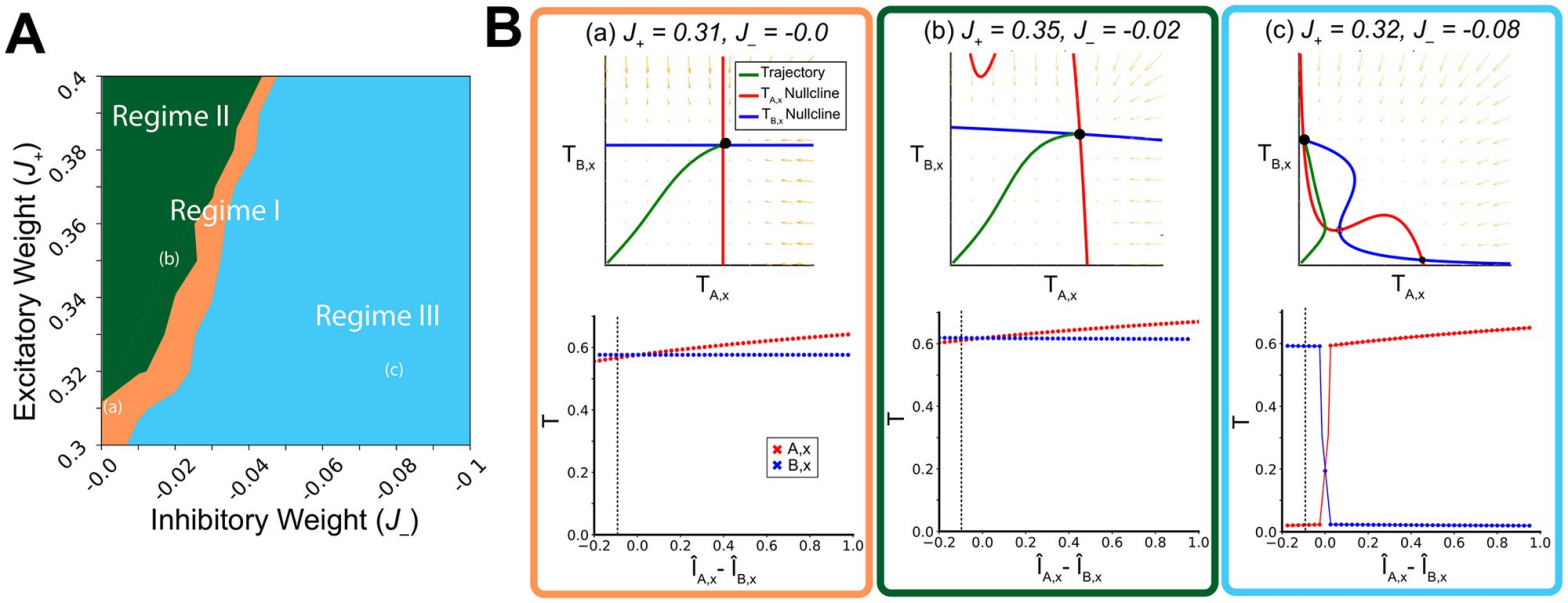
Hierarchical network decision regimes produce linear and nonlinear operations. *I*: input; $\hat{I}$: input normalized by the maximum input value; *T*: the transformed output of an intermediate layer; *J*_+_: excitatory weight; *J*_−_: inhibitory weight; red: choice alternative *A*; blue: choice alternative *B*; green: trajectory from a starting point of (0.06, 0.06); solid circles: stable fixed points; large solid circle: trajectory endpoint. (A) Using fixed point approximations, the E/I tone space can be partitioned according to decision regimes similar to the full model. The color scheme is the same as Fig 2D. (B) Phase portraits and bifurcation diagrams show that regimes III performs a highly-nonlinear winner-take-all operation. The E/I tone is provided both in the heading, and indicated in (A) by the lower-case marker. The top row contains phase portraits produced by a specific set of inputs (*I*_*A*,*x*_ = 20, *I*_*B*,*x*_ = 18). The relative value of the inputs is indicated by the vertical dashed line in the bifurcation diagrams of the second row. To produce the bifurcation diagrams, the input-output of a single attribute value is varied (*I*_*A*,*x*_) while the other (*I*_*B*,*x*_) is fixed. The difference between the normalized fixed (${\hat{I}}_{B,x}$) and the varied value (${\hat{I}}_{A,x}$) is on the X-axis. The *T* value of the endpoints is on the Y-axis. Individual trajectory endpoint values *T*_*A*,*x*_ (red) and *T*_*B*,*x*_ (blue) values are plotted, along with lines joining them when needed to aid visualization.

We then used fixed points to compute indifference curves, similar to those shown in Fig 2C. In Fig 3A, we show the decision-making regimes as computed from the intermediate layer fixed points (as in Fig 2D). Its qualitatively similar form to that of Fig 2D further supports that the fixed points provide a reasonable approximation of the full network.

Confident in the representativeness of the intermediate layer fixed points, we used them to investigate the input-output relationship of various intermediate layer E/I tone configurations. First, we examined individual phase planes from E/I tone configurations in the three regimes (top row of Fig 3B), receiving inputs *I*_*A*,*x*_ = 18 Hz and *I*_*B*,*x*_ = 20 Hz. While the endpoints of of the intermediate transform layer areas in regimes I and II qualitatively maintained the relationship of the input (left and middle of the top row in Fig 3B), regime III performed a highly-nonlinear winner-take-all operation.

To better characterize this operation, we first looked at how the output changes as one input is increased while all else is held constant. We then assessed the how multiple attributes interact as they pass through the intermediate layer.

To investigate how output changes as only one input is varied, we kept one of the attribute inputs (*I*_*B*,*x*_) to an intermediate layer transform area fixed, while increasing the level of the other attribute input (*I*_*A*,*x*_). The resultant *T*_*A*,*x*_ and *T*_*B*,*x*_ for the three regions is shown in the bottom row of Fig 3B. Note that in regimes I and II the constant-input does not change as the magnitude of the variable input increases (Fig 3B, bottom left and middle). In regime III, when the variable input is less than the fixed input, the output-value of the variable input is suppressed; however, when the variable input is greater than the fixed input, the output-value of the constant input is suppressed, while the output-value of the varied input grows approximately linearly (Fig 3B, bottom row on the right). Thus, in regime I and II, each input value passes through the intermediate layer, with a degree of linearity defined by the E/I tone. In regime III, however, a qualitatively different function is implemented such that the smaller input value is suppressed by a max operation.

### Hierarchical network performance relative to the linear network improves under uncertainty

Having characterized the decision-properties in three regimes of the Hierarchical Network and the basic operations performed by under these regimes, we investigated how performance of the Hierarchical Network compares to that of a conventional Linear Network. Uncertainty as to information has been a been widely studied in the context of multi-attribute decision-making, as well as value-based decision-making [23]. Indeed, many real-world decisions are made in environments where the true value of an attribute has a known degree of uncertainty. One could divide the source of an attribute’s known uncertainty into three categories: 1) external representation in the environment, 2) transmission of externally represented values into the brain, 3) internal generation of values within the brain. Case 1 could occur when bargaining for a good in a market, when there is an estimated uncertainty as to whether the value being quoted by the other party is accurate. Case 2 could arise when reading prices on a menu that are listed in a foreign currency; the numbers on the menu are accurate, but there is an estimated uncertainty as to the ability to convert the currency (from Rupees to Yuan) in one’s head. Case 3 could take place when one is assigning value to the amount of happiness a toy will bring for a young niece on her birthday, when there is an estimated uncertainty as to her preferences. While each of these cases involves a unique processing of individual attribute values prior to entering our network models, once they reach the network models they are identical.

Such uncertainty could be incorporated into a model’s attribute weights, or as a separate attribute unto itself. Yet, both approaches would fail to significantly alter performance in environments where the level of uncertainty rises uniformly. For example, if one incorporates uniform environmental uncertainty in attribute weights as the inverse of the variance, all attributes will decrease by the same proportion, thus having negligible effect on the decision process. Similarly, if one incorporates uncertainty as a separate attribute, the same attribute value will contribute to both alternatives; while this may affect the reaction time in a dynamical model, it again will fail to shift the decision strategy.

Instead, a global increase in environmental uncertainty should be addressed with a shift in the underlying function applied to attributes. We therefore investigated performance of our networks in environments where varying degrees of value stochasticity created uniform uncertainty as to the true attribute values.

To control the level of uncertainty in the environment, we added a stochasticity term *η*_*I*_ to each input at the beginning of the trial, independently drawn from a normal distribution centered on 0, with a variance of $\sigma_{\eta_{I}}^{2}$. We then sequentially presented networks with an array of 630 offers, which were translated to firing rates of 10 to 20 Hz, spaced by 2 Hz. The inputs were produced combinatorially, such that on one trial the alternative *A* attributes might be [14 Hz, 12 Hz] and alternative *B* attributes [12 Hz, 18 Hz]. On the next trial, *A* could be composed of [10 Hz, 12 Hz] and *B* [20 Hz, 18 Hz], etc. These choices were presented in blocks, where each block had a fixed $\sigma_{\eta_{I}}^{2}$ that ranged from 0 to 2 Hz. To simplify the analysis, we assumed linear subjective value functions.

**Fig 4:**
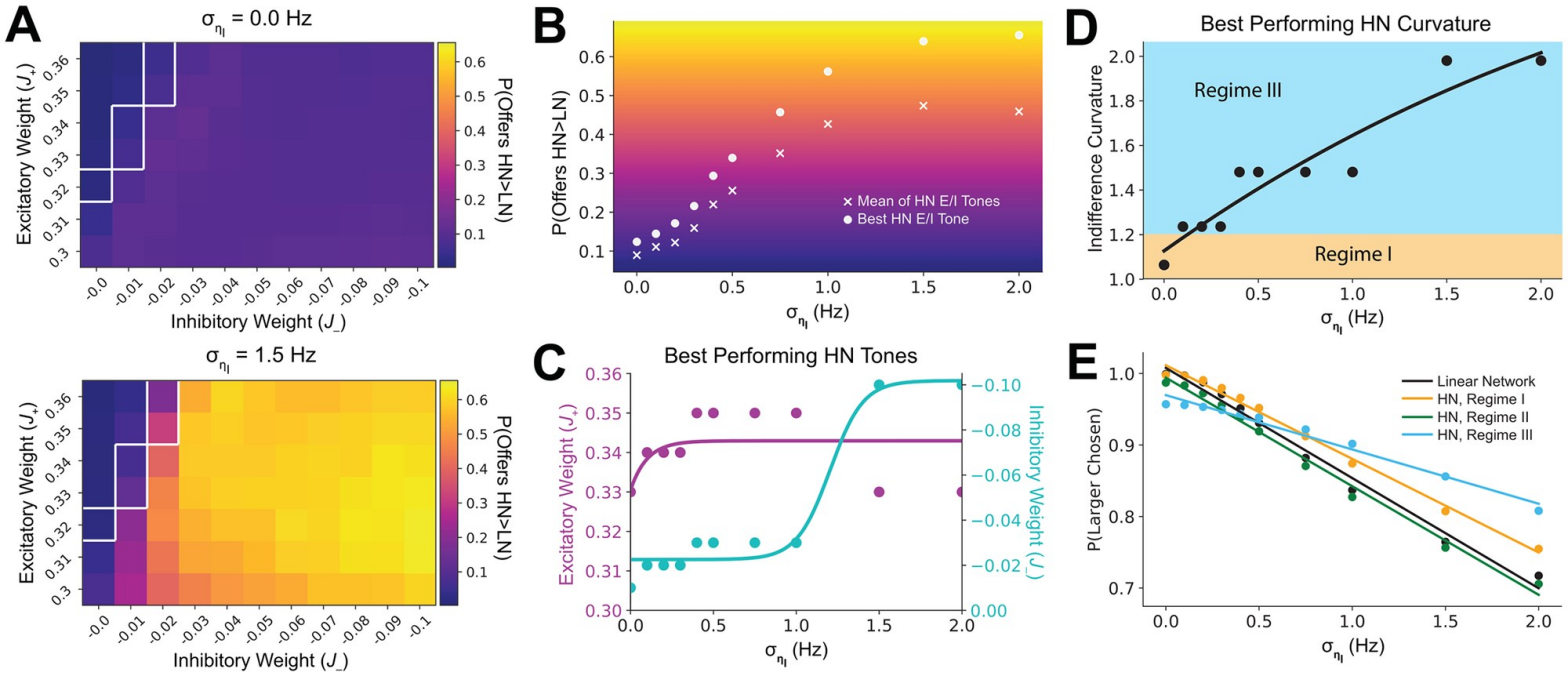
Hierarchical network in regime III outperforms linear network as uncertainty increases. HN: Hierarchical Network; LN: Linear Network; *J*_+_/purple: HN intermediate layer excitatory weight; *J*_−_/turquoise: HN intermediate layer inhibitory weight; $\sigma_{\eta_{I}}$: environmental uncertainty; white outline: regime I. (A) Performance of HN relative to LN improves across offers as uncertainty increases. Networks were presented with 640 unique offers. The proportion of offers for which networks chose the larger combined alternative was measured. Heatmaps show the proportion of offers where the HN is more likely than the LN to chose the larger alternative. HN E/I tone are given on the X-axis and Y-axis respectively. (Top) Relative performance with no environmental uncertainty. (Bottom) Relative performance at high environmental uncertainty. (B) The mean of HN E/I tones and the best HN E/I tone both improve in performance relative to the LN as uncertainty increases. The X-axis indicates the amount of environmental uncertainty, while the Y-axis indicates the proportion of offers the HN displays better performance than the LN. The ‘x’ markers indicate the mean of all E/I tones in (A), while the dots indicate relative performance of the best E/I tone. (C) Best-performing inhibitory tone increases with uncertainty. Excitatory and inhibitory weights are given for the best performing HN intermediate layer E/I tone at each uncertainty level. A sigmoid function was fit to the points. (D) The indifference curvature of HN high-performing E/I tones becomes increasingly concave. The curvature is given as a function of environmental uncertainty for the best-performing E/I tone. (E) Performance of regime III degrades less with uncertainty relative to regimes I and II or the linear network. Values and linear fits are given for the LN, and as well as weight configurations representing the three HN network regimes (regime I: *J*_+_ = 0.34, *J*_−_ = −0.01; regime II: *J*_+_ = 0.36, *J*_−_ = −0.0; regime III: *J*_+_ = 0.32, *J*_−_ = −0.1).

For the Linear Network and each E/I tone configuration of the Hierarchical Network, we first calculated the proportion of trials for each offer where the larger combined alternative value was chosen. We then compared the proportion of offers where the Hierarchical Network was more likely than the Linear Network to select the choice alternative with the larger combined value. We found when there is no environmental uncertainty, the Linear Network out-performs the Hierarchical Network on the vast majority of offers (Fig 4A, top). However, at a high level of environmental uncertainty, the proportion of offers increases where the Hierarchical Network outperforms the Linear Network (Fig 4A, bottom). This improvement in relative performance as uncertainty increases is observed both in the mean relative performance of all E/I tones, and in the relative performance of the best E/I tone (Fig 4B). When examining the best performing E/I tones, we found that the excitatory tone remains relatively constant, while the inhibitory tone increases (Fig 4C). Crucially, this shift in E/I tone behaviorally manifested in a shift of observable indifference curvature, where concavity increases along with uncertainty (Fig 4D).

To better understand this transition in the indifference curvature, we examined the overall proportion of trials where the larger offer was chosen as a function of uncertainty for the Linear Network and several E/I tones from Hierarchical Network regimes (Fig 4E). Though the regime III E/I tones under-perform at low uncertainty, they display less degradation as uncertainty increases, eventually crossing that of the Linear Network and regime I. Thus, the Hierarchical Network displays distinct decision regimes that maximize choice performance under a variety of environmental uncertainty levels. Furthermore, the shift in regimes with uncertainty is primarily achieved through increasing inhibitory tone, and is behaviorally differentiable.

### Hierarchical network regime III selectively increases offer separability and attends to the salience of attribute differences

Having established that different decision-making regimes maximize reward at different uncertainty levels, as well as how the network produces these regimes, the natural question arises as to why this occurs.

Given the results of the fixed point analysis (as shown in the right column of Fig 3B), regime III appears to be performing a winner-take-all function, with the larger of the attribute input values transmitted and the smaller quashed. This resembles max operations, where the final area operates on the maximum within-attribute values of the intermediate transform areas. To gain intuition as to how the Hierarchical Network could out-perform the Linear Network, we compared a simplified linear combination to one involving attribute-level max operations.

**Fig 5:**
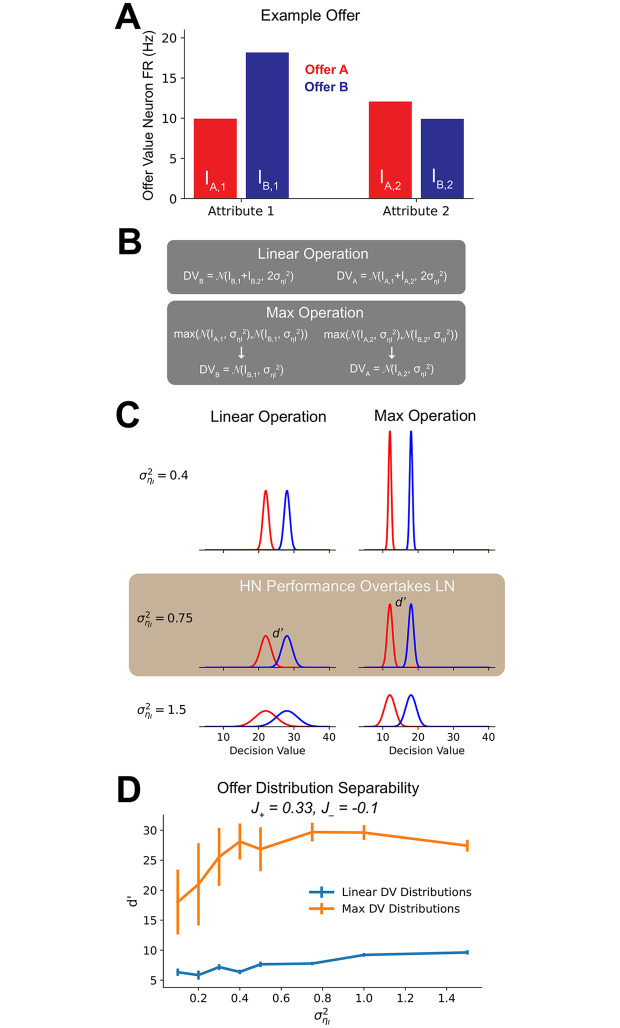
Hierarchical network regime III winner-take-all operation increases offer separability. (A) Example offer where Regime III improves in relative performance as uncertainty increases. (B) Linear and max operations used to calculate decision value distributions for the offer shown in (A). In a linear operation, the mean and variance of both attribute are used to calculate distribution from which the final offer value is drawn. The max operation first selects the larger within-attribute alternative, which then becomes the decision value distribution. (C) Decision value distributions produced by the max operation maintain greater separability as uncertainty increases. Distributions are shown for the decision values calculated in (B) using $\sigma_{\eta_{I}}^{2}=0.4$ (top row), $\sigma_{\eta_{I}}^{2}=0.75$ (middle row) and $\sigma_{\eta_{I}}^{2}=1.5$ (bottom row). In the left column are results for the linear operation, and in the right from the max operation. (D) For those offers where regime III performance (*J*_+_ = 0.35, *J*_−_ = −0.1) exceeds that of the LN, a max operation provides greater separability between decision value distributions. For offers that transitioned to better HN performance, at each uncertainty level we calculated the *d*′ of decision value distributions produced by the linear operation and the max operation. Shown are the mean and standard error of the *d*′s.

For the linear operation, the final decision value was simply a linear sum of attribute distributions. The max operation, however, first took a maximum of the attribute distributions while at the attribute level. An example offer is given in Fig 5A, and the set of equations in Fig 5B.

We then examined the set of offers where, as uncertainty increased, the Hierarchical Network began to out-perform the Linear Network (indicated in Fig 4B). When the transition in performance occurred, the Linear Operation decision value distributions began to overlap, while the Max Operation decision value distributions remained separable (Fig 5C). We quantified this by calculating the mean and standard error of the *d*′ values for the linear and max operation distributions at each uncertainty level (Fig 5D). The *d*′ value quantifies the separability of two distributions. As anticipated, for those offers where Hierarchical Network performance overtook that of the Linear Network, the max operation produced significantly higher separability between the offer distributions.

**Fig 6:**
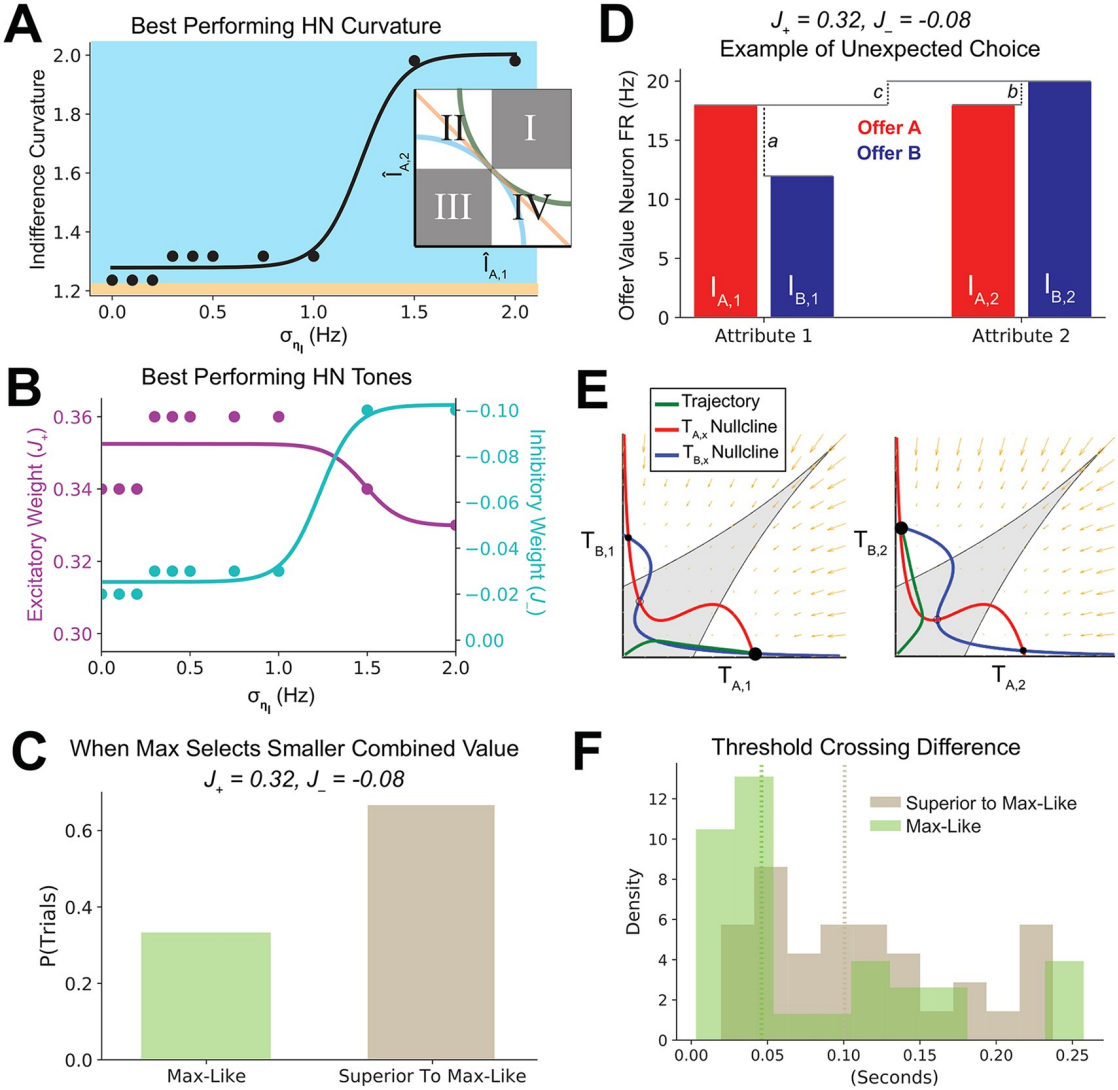
Hierarchical network regime III preferentially attends to attributes with larger cross-alternative differences. Lime green: choice behavior matching that of a max operation; khaki: choice behavior choosing higher combined value; gray: region prior to threshold crossing. (Inset A) Offers were selected from quadrants of the indifference curve offers plane where the three regimes display differentiable choice behavior. Quadrants II and IV, from which offers were selected, is given in white. Example indifference curves from the three regimes are shown. (A) As in Fig 4E, the best performing indifference curvature becomes more concave as environmental uncertainty increases. (B) As in Fig 4C, the best performing E/I tone as a function of environmental uncertainty shows increasing inhibitory tone. (C) A regime III E/I tone performs better than expected of a pure sequential-max network. (D) An example offer where a pure max operation would select the smaller combined value, but the regime III area selects the larger combined value. (E) Phase portraits from the values in (D), showing that the regime III areas still perform a winner-take-all function. (F) On trials where the regime III area out-performed a pure max operation, the area with greater attribute value differences has population firing rates that more quickly diverged (leaving the gray area of (E)). Shown is a histogram of firing rate divergence time differences between attribute areas for trials when the network performed as predicted by a max operation, and a histogram of trials where it performed superior to it.

We then asked how the distribution of offer attribute values affected the relative performance of the decision regimes. Indeed, if one simply used the max operation as a heuristic on a pure combinatorial sampling of offers, one would correctly select the largest choice alternative on 88.9% of trials. We therefore focused on offers that could maximally differentiate the three Hierarchical Network regimes. Offers used to assess indifference curves can be divided into four quadrants (Fig 6A, inset). In quadrants I and III, all three regimes are predicted to choose the same alternative. Quadrants II and IV, however the three regimes predict differentiable choice behavior. For example, a pure max operation would choose the smaller alternative for 50% of offers. We thus sampled 91 offers from quadrants II and IV (see Materials and methods). Surprisingly, in the Hierarchical Network intermediate layer, the best-performing E/I tone configurations again became increasingly concave as uncertainty increased (Fig 6A). Furthermore, the E/I tone displayed a similar trend of relatively stable excitation, with a monotonic increase in inhibition (Fig 6B). If regime III were performing a pure max operation, this would not be observed.

To interrogate why the network was performing better than expected, we focused on the subset of the 640 offers where a pure max operation would choose the smaller choice alternative. We selected a single regime III E/I tone configuration (*J*_+_ = 0.32nA, *J*_−_ = −0.02 nA), then calculated the proportion of trials where the network performs as predicted of a max operation, as well as the proportion of trials where the network is superior to predictions. On 66.7% of trials, the network was superior to the pure max operation (Fig 6C).

We then examined a typical trial where the Hierarchical Network performed better than expected (Fig 6D). On this trial, the combined value of alternative *A* attributes was greater than the combined value of alternative *B* attributes (*I*_*A*,1_ + *I*_*A*,2_ > *I*_*B*,1_ + *I*_*B*,2_), but *I*_*A*,1_ > *I*_*B*,1_ and *I*_*A*,2_ < *I*_*B*,2_. Thus, in a pure sequential-max operation, the final choice would be between *I*_*A*,1_ and *I*_*B*,2_. As *I*_*A*,1_ < *I*_*B*,2_, a pure sequential-max operation would chose alternative *B*, which is the smaller combined alternative. However, the regime III networks chose alternative *A*. This could be achieved if, rather than just attending to the largest attribute value, the network considered the magnitude of the difference within-attributes. For example, if the network attended to the fact *a* ≫ *b*, it could arrive at choice alternative *A*, which is the higher valued offer.

To achieve this more sophisticated operation, the network may simply on select offers perform an input-transformation different from winner-take-all. To test this possibility, we examined the phase portraits produced with these inputs by intermediate layer transform areas (Fig 6E). Rather than selectively shifting to a different operation, both areas consistently performed a winner-take-all function.

We then asked if the dynamics of the network were accomplishing a more sophisticated nonlinear computation. To do this, we set a firing-rate threshold difference of 12 Hz (gray in Fig 6E) within each attribute-specific intermediate layer transform area (see Materials and methods). We presented the network with offers where a sequential-max operation would choose the smaller choice alternative (Fig 6C). For these offers, we measured in each area the time at which the threshold was crossed. Finally, we compared the time of threshold crossing between attribute-specific areas. This produced a “threshold crossing difference,” for each set of choice alternatives. The difference in threshold crossing was consistently larger in those cases where the network performed better than expected (Fig 6F). Mechanistically, the earlier the threshold is crossed in an intermediate area, the more it biases the competition in the final area [62]. This indicated that the network codes the magnitude of attribute difference, and does so using time dynamics.

Thus, advantageous performance in regime III was achieved through a combination of increasing offer separability, and selective attention to the salience of attributes with a larger cross-alternative difference.

### Readout from intermediate layer transform areas is accurate, and under uncertainty improves relative to the final area

The use of time dynamics by the intermediate layer transform areas to perform sophisticated nonlinear computations suggests more can be learned by examining the readout from lower areas in the hierarchy. Experimentally, decision signals can be read out from numerous brain areas. This has prompted a larger conversation as to where decisions actually take place, how brain areas contribute to decision-making, and the relevance of experimental findings [9, 10, 10, 19–21, 63].

**Fig 7:**
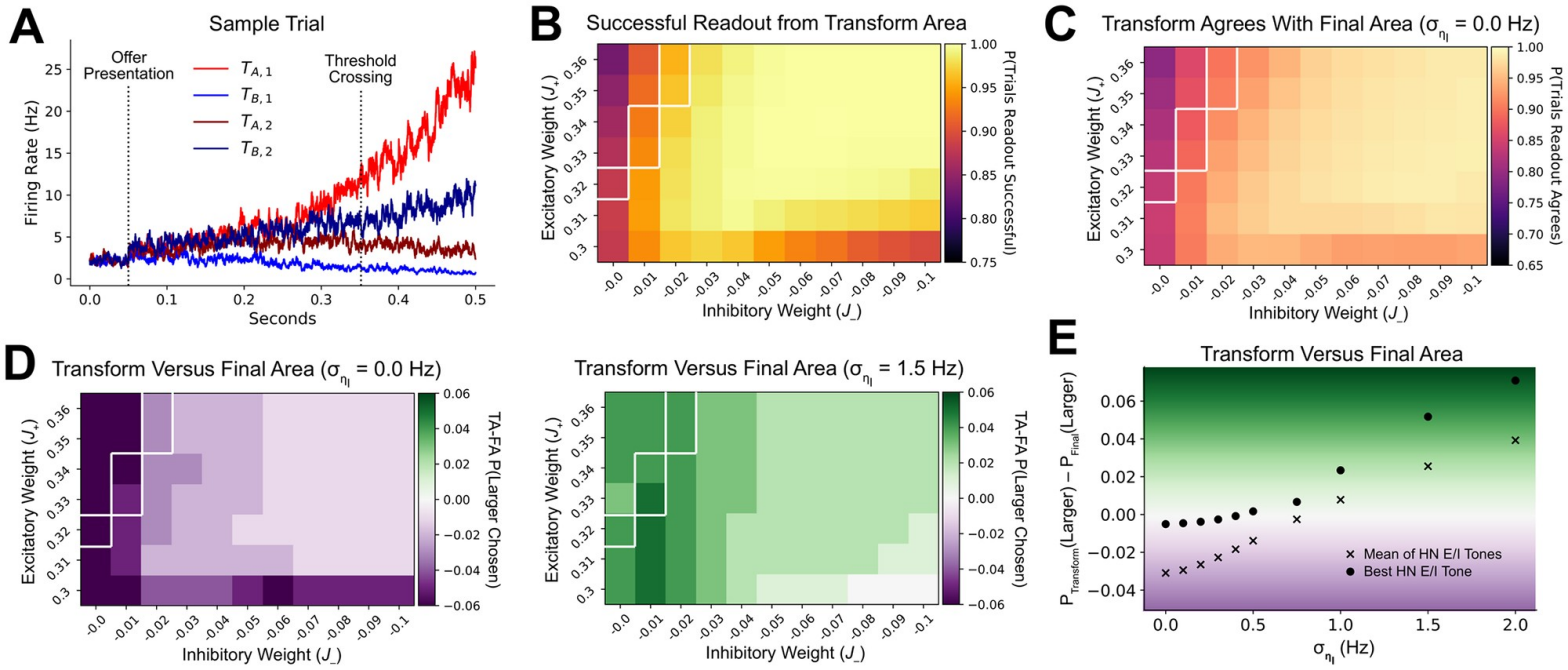
Intermediate layer areas provide accurate readout. TA: intermediate layer transform areas; FA: final area; light red: firing rate for population *T*_*A*,1_; light blue: *T*_*B*,1_; dark red: *T*_*A*,2_; dark blue: *T*_*B*,2_. (A) A decision is read out when the firing rate difference within an area exceeds a threshold of 12 Hz. Inputs and E/I tone as in Fig 6D and 6E. Vertical dotted lines indicate The onset of the offer and the time at which the firing-rate difference crosses the threshold. (B) Decisions are consistently read out from regime III. Heatmap shows the proportion of trials for different HN E/I tones where the intermediate layer TAs crosses the firing rate threshold before the FA reaches a decision. (C) Regime III TA readout agrees with FA. Proportion of trials (at zero environmental uncertainty) where the readout from the intermediate layer TA agrees with that of the FA. (D) TA readout is more likely than FA to select higher valued option as uncertainty increases. The relative performance at choosing the larger offer by the TA and FA at low (left) and high (right) environmental uncertainty. Green indicates superior performance by the TA, while purple indicates superior performance by the FA. (E) Summary plot of performance differences between the TA and FA as a function of environmental uncertainty for the mean of all TA E/I tones, as well as the best performing TA E/I tone.

To read out from intermediate layer transform areas, we extended our previous threshold analysis, such that the choice was determined by the first population to exceed the threshold difference within an area. An example trial is given in Fig 7A. Importantly, we assumed that the network decision was still being determined by the final area, meaning that our readout from intermediate layer transform areas was similar to that done during an electrophysiology or imaging experiment. Because of this, if no intermediate layer transform area crossed the threshold prior to the final area crossing its threshold, we classified that trial as a failed readout.

We first examined the proportion of trials for which a successful transform-area readout was obtained, finding that regime III areas with high E/I tone were successful on nearly every trial (Fig 7B). This was due to high recurrent excitation driving an increase in firing rate, while high inhibition drove a resolution of the competition. We further found that at low uncertainty, readout from many E/I tones in regime III agreed on nearly every trial with that of the final area (Fig 7C). This has a crucial experimental interpretation: even with the decision determined by a later area, it is possible to readout that decision with near 100% accuracy from an earlier area; furthermore, the early area readout can be obtained prior to that recorded from the final area. Thus, the mere presence of a decision signal and its relative timing cannot definitively be used to indicate the role of a brain area in decision-making.

Experimentally, neurons are occasionally identified that are more selective than the actual subject for stimulus value or motion coherence. Explanations have been proposed that such neurons provide specialized signals, or are an analytical artifact [64, 65]. The relatively simple feed-forward, mean-field architecture of the Hierarchical Network provides another avenue of investigation for this phenomenon. To do so, we compared the accuracy of readout from an intermediate layer transform area with readout from the final area. This was done by first computing the proportion of trials in each layer for which the larger choice alternative was selected; then, we subtracted *P(Larger Chosen)* by the final area from *P(Larger Chosen)* by the transform area. We found that in the absence of uncertainty, readout from the final area is always better than that of the intermediate layer, though the high-performing E/I tone configurations from Fig 7B and 7C comes close to matching the final area (Fig 7D, left). However, at high uncertainty, readout from the intermediate layer transform areas exceeds that of the final area (Fig 7D, right). The difference is summarized in Fig 7E for the mean of all E/I tones, and the best E/I tone. The improvement in relative performance was largely driven by regime I and II E/I tone configurations (as seen in the heatmap of Fig 7D, right). These regions have less inhibition to slow ramping activity. Thus, attribute pairs with larger differences between offers more quickly crossed the FR difference threshold; In effect, these regions provided an alternative mechanism for the filtering of less-informative attribute information in situations of high uncertainty. From this result arises two important experimental implications: first, it is possible to obtain superior readout from lower-hierarchy areas, even in a feed-forward network without specialized functions; second, this readout can occur in regimes where attribute value information is linearly transmitted.

### Choice patterns of model with restricted inhibitory tone differs from control as environment uncertainty increases

Having shown that the Hierarchical Network can adopt multiple decision-making regimes, that the regimes maximize performance in different environments, and how E/I tone defines regime-specific attribute transforms, we next hypothesized a framework where E/I tone can be modulated according to the degree of environmental uncertainty. We then applied this framework to investigate altered decision-making in conditions with limits on inhibitory tone.

The theory of E/I imbalance is one of the leading biophysical frameworks for understanding psychiatric disorders such as ASD and SCZ. Yet given the vast number of manners in which E/I imbalance can arise, it is important to identify meaningful mechanisms on which specific etiologies converge [24]. Though the biological details in each condition likely differ (reduced PV interneuron expression in ASD, and reduced excitation of inhibitory cells in SCZ), and they appear to involve unique sets of brain areas (targeted areas in ASD, and widespread alterations in SCZ), both conditions show evidence of a shared mechanism involved reduced inhibitory tone [25, 28, 29]. While reduced inhibitory tone has been shown to impact visual processing in both ASD and SCZ, far less work has been done on its cognitive implications [25–27]. This is all the more important as the symptomology of both disorders is less characterized by basic sensory deficits than altered cognition.

In addition to restricted inhibitory tone in SCZ and ASZ, there is evidence for a general intolerance of uncertainty [66–69], and altered performance with environmental uncertainty [70–73]. Furthermore, differences have been found in multi-attribute decision tasks [74–76].

Therefore, to investigate novel connections between altered cognition and abnormal E/I tone, we tested variants of the Hierarchical Network on a multi-attribute decision task with uniform environmental uncertainty as to the true value of attributes. Sessions were composed of blocks of trials, during which the uncertainty level of offers was stable. At the beginning of each block, the network was given the level of environmental uncertainty. It then chose between offers composed of two attributes (assumed to be of equal subjective value), with the goal of accumulating the greatest total reward.

The E/I tone of a brain area is not necessarily fixed. Indeed, there are several mechanisms by which the tone can be temporarily modulated [77–79]. The cholinergic diffuse modulatory system, in particular, provides a plausible mechanism for the source of E/I tone shift due to expected environmental uncertainty [80–87].

**Fig 8:**
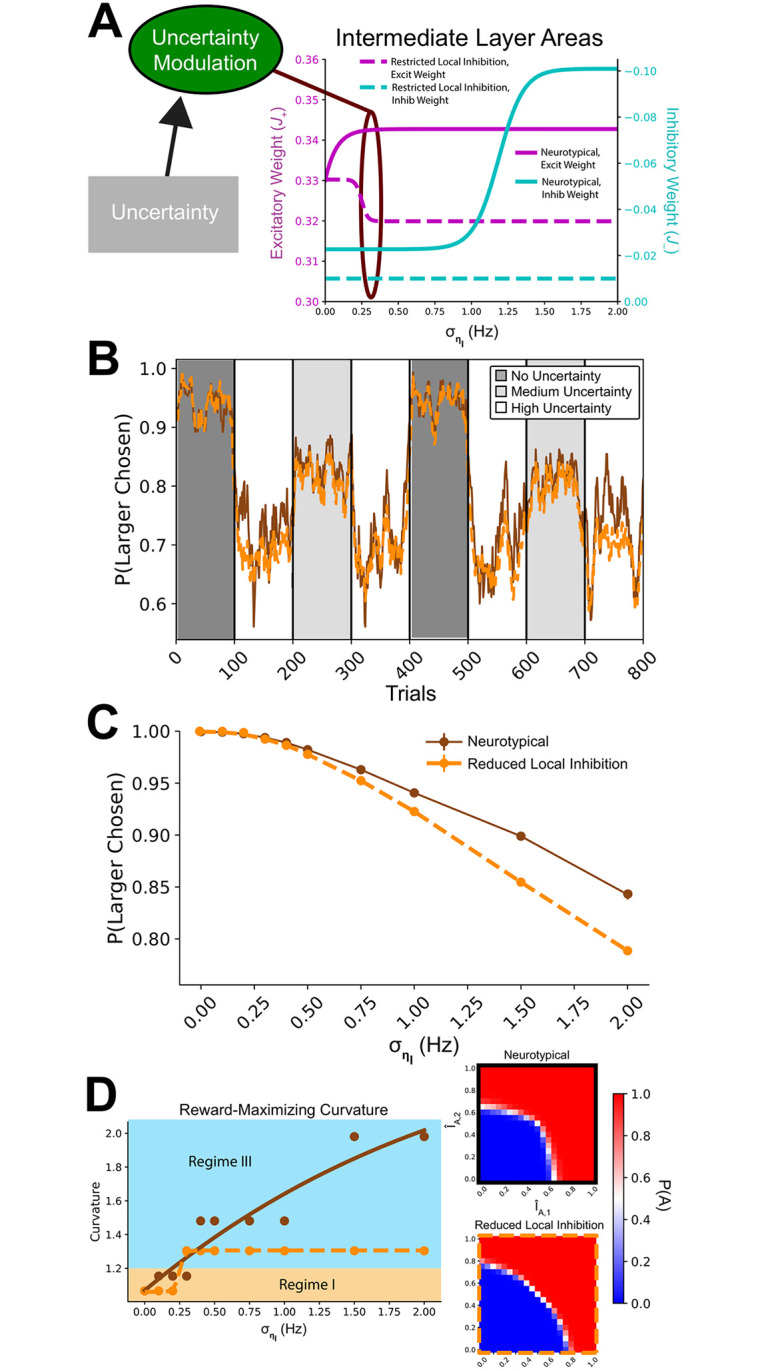
Task performance under uncertainty differs between neurotypical and restricted local inhibition models. (A) A module was added (forest green) that modulates the intermediate layer E/I tone according to the uncertainty level. The E/I tone were determined using continuous functions, similar to those shown in Figs 4C and 6B. In the restricted inhibitory tone model, inhibition was limited to −0.01. (B) A single session of the simulation, where the X-axis is trial number, and Y-axis is the proportion of trials where the models choose the larger option. (C) Performance between the models diverges as uncertainty increases. The proportion of the trials where the larger alternative was chose is given as a function of uncertainty for both models. (D) The optimal indifference curvature differs between models as uncertainty increases. To the right are example indifference curves for neurotypical (top) and restricted local inhibition (bottom) models at their maximum level of concavity. Input values ($\hat{I}$) are normalized by the reference choice alternative values’ linear sum.

To allow the network to adapt to uncertainty levels, we added a module that used a continuous function to identify the optimal intermediate layer E/I tone for a given environmental uncertainty level, then could shift weights in the attribute-specific intermediate layers to that tone (Fig 8A). The control model was able to explore the full range of possible weights. The model with restricted local inhibition was implemented by limiting the possible inhibitory tone in intermediate attribute-specific areas to a maximum magnitude of −0.01 nA.

Sessions were composed of trial blocks with variable levels of uncertainty. Observing performance in a single session, we could already see a separation of performance levels during high-uncertainty blocks (Fig 8B). Analyzing *P(Larger Chosen)* as a function of uncertainty, we found the restricted local inhibition model did not differ from the neurotypical model when the uncertainty level was low, but began to diverge as uncertainty increased (Fig 8C). We also examined the change in the indifference curve as a function of uncertainty, finding a ceiling to the concavity of the restricted local inhibition model’s decision behavior (Fig 8D). Thus, the models make two strong experimental predictions: 1) performance will be identical at low uncertainty, while subjects with restricted local inhibition will show a greater fall-off in total reward as uncertainty increases; and 2) at high uncertainty, subjects with restricted local inhibition will continue to show more linear-weighting of attribute values than neurotypicals, who shift to a nonlinear strategy of weighting the attribute by their difference.

## Discussion

We show that a feed-forward hierarchical network performing a multi-attribute decision task can adopt distinct behavioral regimes according to the E/I tone of intermediate layers. Moreover, the sophisticated decisions enabled by nonlinear transforms of the intermediate layer become increasingly advantageous in environments where there is uncertainty as to the true value of attributes. The behavioral regimes are largely dictated by inhibition, providing specific predictions of how performance will differ between neurotypicals and subjects with restrictions on inhibitory tone. A list of neurophysiology and behavioral predictions are given in Table 1.

**Table 1:** Experimental predictions as known attribute uncertainty increases.

**Neurophysiology Predictions**
1) Firing rates in areas with integrated decision signals will shift from representing an integration of all offer attributes to representing an integration of attributes with greater salience.
2) Firing rates representing individual attributes will shift from a linear response for all attribute values to reflecting a more winner-take-all operation.
3) The timescales of neural responses will slow (due to an increase in inhibition).
4) In recordings from brain areas upstream of a decision, readout performance may improve relative to the actual decisions.

**Behavioral Predictions**
1) Subjects maximizing reward will increasingly base decisions on attributes with a larger difference between offers. This can manifest as a shift in the indifference curve from convex, to linear, to concave.
2) Subjects with restrictions on inhibitory tone will display less flexibility in strategy shifting.

Several electrophysiology studies have specifically investigated two-alternative multi-attribute tasks, while recording from down-stream areas relevant for decisions. Reward magnitude, probability, information value, social hierarchy and juice type have all been found to be represented in areas like the orbitofrontal cortex (OFC) or anterior cingulate cortex (ACC), and in a manner where all attribute signals were available for decision-making [1–3, 8, 14, 18, 88–93]. Moreover, the integration of value and action signals has been observed in the dlPFC, similar to that occurring in our models’ final area [22]. However, it is important to recognize that the stimuli used to signify attribute information in these studies were unambiguous, and that NHP subjects were over-trained on the use of attributes to optimize reward. Thus, these studies all take place in the “low uncertainty,” range of our results. A quasi-linear attribute value transmission is therefore expected. However, in a study where NHPs chose between two options, each composed of symbols whose combined value was a linear sum, they exhibited increased weighting of the larger attribute in early sessions, and shifted towards a more linear summation later in later sessions [94]. This is exactly the strategy predicted by our model, as the NHP’s uncertainty regarding attribute values would decrease over the course of learning. Our results suggest that if environmental uncertainty is systematically introduced, one should observe in areas such as the OFC or ACC an over-representation of attribute values that have a larger difference, and that the activity will correlate with animal behavior. This is a distinct prediction, highly feasible for experimentation.

Our results on readout from intermediate layers also provide several notes of caution for the interpretation of experimental findings. First, simply because recordings from a brain area provide a consistent, early readout of decision behavior does not mean the area is located in a position of the hierarchy with access to all information integrated in a decision process. Second, even a simple feed-forward network can be configured such that the key decision computations take place in different areas (the final area for regime I and the transform areas in regime III). The brain is a highly recurrent system, where each area is capable of sophisticated operations and can be uniquely recruited based on task demands. Thus, key neural computations during a decision process are even less likely to be consistently localized in a single brain area. Finally, under certain conditions intermediate layer areas selected the higher-valued offer more consistently than the area that determined the choice of the network. Rather than being a unique feature, similar experimental observations may similarly be an emergent property of a dynamic decision process.

In our final simulations, we hypothesized a network where the E/I tone of an area can be shifted based on the task. Though the biology of how E/I tone of specific areas can be tuned according to task demands remains to be fully characterized by experiments, diffuse modulatory neurotransmitter systems provide the most plausible mechanism. Dopamine, norepinephrine, serotonin, nitric oxide and acetylcholine (ACh) have all been shown to influence the E/I tone of the cells that they target [79]. These influences can be complex, with cell-type specific shifts in excitation or inhibition implemented via a variety of mechanisms (metabotropic receptors, ion channels, etc.). The ACh system in particular displays the area-specific targeting [95], the modulation of inhibitory tone [83–87, 96–102] and the activity associated with known uncertainty [80, 82, 103–105] that is required by our model. However, it remains to be investigated precisely how the brain can identify an task-optimal E/I tone for a circuit and then shift the circuit to that value.

Our work makes additional strong predictions for the behavior of subjects with inhibitory tone dysfunction performing a multi-attribute decision task under uncertainty. There are a number of existing experimental results in the fields of SCZ and ASD research that suggest our predictions are reasonable and worth investigating. Several studies show that when attribute information is clear, performance between subjects with these conditions and neurotypicals is identical, while it differs in the face of noise or uncertainty. Dot field motion tasks, in particular, have been used to study these questions in subjects with SCZ or ASD. Subjects with SCZ tend to differ from neurotypicals at all levels of coherence [72, 73]. The general finding in ASD has been that when coherence of the moving dot field is high (uncertainty is low), subjects with ASD and neurotypicals show similar performance [70, 71, 75]. Indeed, in detection of velocity, and some coherence studies, subjects with ASD outperform neurotypicals [106]. However, when coherence levels decrease (uncertainty increases), subjects with ASD consistently show significantly greater decreases in performance. Similar effects of environmental uncertainty have been shown in subjects with either condition performing multi-attribute integration [74–76]. Our results suggest a plausible mechanism for the effects observed in prior studies, while also proposing a specific experiment with falsifiable findings.

In this paper, we used a feed-forward hierarchical neural network of cascading nonlinearities to investigate how biophysical properties, such as parallel structure and E/I tone, shape sophisticated strategies during multi-attribute, multi-alternative decisions. This investigation produced strong predictions for electrophysiology experiments, as well as experiments involving human behavior. Our work thus suggests a new avenue for research connecting neural circuits to multi-attribute economic choice and to deficits associated with restrictions on inhibitory tone.

## Conclusion

In this paper, we used a feed-forward hierarchical neural network of cascading nonlinearities to investigate how biophysical properties, such as parallel structure and E/I tone, shape sophisticated strategies during multi-attribute, multi-alternative decisions. This investigation produced strong predictions for electrophysiology experiments, as well as experiments involving human behavior. Our work thus suggests a new avenue for research connecting neural circuits to multi-attribute economic choice and to deficits associated with restrictions on inhibitory tone.
